# Exposure–response relationships for personal exposure to fine particulate matter (PM_2·5_), carbon monoxide, and black carbon and birthweight: an observational analysis of the multicountry Household Air Pollution Intervention Network (HAPIN) trial

**DOI:** 10.1016/S2542-5196(23)00052-9

**Published:** 2023-05-08

**Authors:** Kalpana Balakrishnan, Kyle Steenland, Thomas Clasen, Howard Chang, Michael Johnson, Ajay Pillarisetti, Wenlu Ye, Luke P Naeher, Anaite Diaz-Artiga, John P McCracken, Lisa M Thompson, Ghislaine Rosa, Miles A Kirby, Gurusamy Thangavel, Sankar Sambandam, Krishnendu Mukhopadhyay, Naveen Puttaswamy, Vigneswari Aravindalochanan, Sarada Garg, Florien Ndagijimana, Stella Hartinger, Lindsay J Underhill, Katherine A Kearns, Devan Campbell, Jacob Kremer, Lance Waller, Shirin Jabbarzadeh, Jiantong Wang, Yunyun Chen, Joshua Rosenthal, Ashlinn Quinn, Aris T Papageorghiou, Usha Ramakrishnan, Penelope P Howards, William Checkley, Jennifer L Peel, HAPIN Investigators

**Affiliations:** a Department of Environmental Health Engineering, ICMR Center for Advanced Research on Air Quality, Climate and Health, Sri Ramachandra Institute for Higher Education and Research (Deemed University), Chennai, India; b Gangarosa Department of Environmental Health, Emory University, Atlanta, GA, USA; c Department of Biostatistics and Bioinformatics, Emory University, Atlanta, GA, USA; d Hubert Department of Global Health, Emory University, Atlanta, GA, USA; e Department of Epidemiology, Emory University, Atlanta, GA, USA; f Rollins School of Public Health and Nell Hodgson Woodruff School of Nursing, Emory University, Atlanta, GA, USA; g Berkeley Air Monitoring Group, Berkeley, CA, USA; h Division of Environmental Health Sciences, School of Public Health, University of California, Berkeley, CA, USA; i Department of Environmental Health Sciences, University of Georgia, Athens, GA, USA; j Center for Health Studies, Universidad del Valle de Guatemala, Guatemala City, Guatemala; k Department of Infectious and Tropical Diseases, London School of Hygiene & Tropical Medicine, London, UK; l Department of Global Health and Population, Harvard T H Chan School of Public Health, Harvard University, Boston, MA, USA; m Eagle Research Centre, Kigali, Rwanda; n Division of Pulmonary and Critical Care, School of Medicine and Center for Global Non-Communicable Disease Research and Training, Johns Hopkins University, Baltimore, MD, USA; o Cardiovascular Division, Washington University School of Medicine, St Louis, MO, USA; p Division of Epidemiology and Population Studies, Fogarty International Center, National Institutes of Health, Bethesda, MD, USA; q Nuffield Department of Women's and Reproductive Health, University of Oxford, Oxford, UK; r Department of Environmental and Radiological Health Sciences, Colorado State University, Fort Collins, CO, USA

## Abstract

**Background:**

Household air pollution (HAP) from solid fuel use is associated with adverse birth outcomes, but data for exposure–response relationships are scarce. We examined associations between HAP exposures and birthweight in rural Guatemala, India, Peru, and Rwanda during the Household Air Pollution Intervention Network (HAPIN) trial.

**Methods:**

The HAPIN trial recruited pregnant women (9–<20 weeks of gestation) in rural Guatemala, India, Peru, and Rwanda and randomly allocated them to receive a liquefied petroleum gas stove or not (ie, and continue to use biomass fuel). The primary outcomes were birthweight, length-for-age, severe pneumonia, and maternal systolic blood pressure. In this exposure–response subanalysis, we measured 24-h personal exposures to PM_2·5_, carbon monoxide, and black carbon once pre-intervention (baseline) and twice post-intervention (at 24–28 weeks and 32–36 weeks of gestation), as well as birthweight within 24 h of birth. We examined the relationship between the average prenatal exposure and birthweight or weight-for-gestational age *Z* scores using multivariate-regression models, controlling for the mother's age, nulliparity, diet diversity, food insecurity, BMI, the mother's education, neonate sex, haemoglobin, second-hand smoke, and geographical indicator for randomisation strata.

**Findings:**

Between March, 2018, and February, 2020, 3200 pregnant women were recruited. An interquartile increase in the average prenatal exposure to PM_2·5_ (74·5 μg/m^3^) was associated with a reduction in birthweight and gestational age *Z* scores (birthweight: –14·8 g [95% CI –28·7 to –0·8]; gestational age *Z* scores: –0·03 [–0·06 to 0·00]), as was an interquartile increase in black carbon (7·3 μg/m^3^; –21·9 g [–37·7 to –6·1]; –0·05 [–0·08 to –0·01]). Carbon monoxide exposure was not associated with these outcomes (1·7; –3·1 [–12·1 to 5·8]; –0·003 [–0·023 to 0·017]).

**Interpretation:**

Continuing efforts are needed to reduce HAP exposure alongside other drivers of low birthweight in low-income and middle-income countries.

**Funding:**

US National Institutes of Health (1UM1HL134590) and the Bill & Melinda Gates Foundation (OPP1131279).

## Introduction

Household air pollution (HAP) exposures from the use of solid cooking fuels such as wood, coal, charcoal, dung, and agricultural residues are a leading risk factor for ill health in low-income and middle-income countries (LMICs), accounting globally for an estimated 2·3 million premature deaths annually and 91·5 million disability-adjusted life-years.^1^ Systematic reviews have summarised the evidence for an association between HAP exposure and adverse health effects, including child pneumonia, chronic obstructive lung disease, lung cancer, and cataracts.^2^ Few studies or reviews have focused on adverse perinatal outcomes including low birthweight.^3^, ^4^, ^5^, ^6^

LMICs bear a disproportionate share of low birthweight (defined as <2500 g regardless of gestational age), accounting for nearly 91% of the global burden.^7^ The aetiology of low birthweight is complex, and despite ongoing efforts to address known risk factors such as maternal malnutrition, malaria, and smoking,^8^ progress has been slow towards the ambitious global nutrition target of a 30% reduction of low birthweight by 2025.^7^ As nearly 3·8 billon people worldwide rely on solid fuels,^9^ a strengthened understanding of the relationship between HAP and low birthweight would be extremely valuable for prioritising efforts to decrease HAP exposures during pregnancy to improve birth outcomes.

**Research in context**

**Evidence before this study**

Household air pollution (HAP) exposures from the use of solid cooking fuels such as wood, coal, charcoal, dung, and agricultural residues are a leading risk factor for ill health in low-income and middle-income countries (LMICs), accounting for an estimated 2·3 million premature deaths annually and 91·5 million disability-adjusted life-years. Several systematic reviews have summarised the evidence for associations between HAP exposures and adverse health effects, including child pneumonia, chronic obstructive lung disease, lung cancer, and cataracts but few have focused on adverse perinatal outcomes including low birthweight. Furthermore, most previous studies examining HAP exposure related health effects have used categorical indicators of exposure, based on primary fuel use. Only three HAP studies to date have reported quantitative exposure–response relationships for birthweight and PM_2·5_ or carbon monoxide and none have examined associations with black carbon. Most studies also did not rely on longitudinal personal exposures during pregnancy or separate the direct effects of these pollutants on birthweight from that mediated through gestational age. Quantitative exposure–response relationships for HAP-associated exposures and birthweight thus remain poorly characterised.

**Added value of this study**

The Household Air Pollution Intervention Network (HAPIN) trial is among the largest household energy intervention trials directed at reducing HAP exposures for pregnant women and their neonates. The study provides one of the largest and most diverse datasets on pregnancy period 24-h personal exposures for PM_2·5_, black carbon, and carbon monoxide, together with measurements of birthweight and ultrasound-assessed gestational age from rural communities in four countries (Guatemala, India, Peru, and Rwanda). Furthermore, primary information on multiple confounders or strong risk factors for birthweight allowed the development of robust models to estimate the strength and shape of exposure–response relationships. The study provides some of the first estimates of exposure–response relationships for gestational black carbon exposures from HAP and birthweight as well as exposure–response relationships for PM_2·5_, black carbon, and carbon monoxide exposures with weight-for-gestational-age *Z* scores. As the results are consistent across four diverse countries, they are widely generalisable. The personal exposure estimates for pregnant women also provide some of the largest numbers of measurements to the WHO global household air pollution database.

**Implications of all the available evidence**

The study provides important new information about exposure–response relationships for gestational HAP exposures and birthweight as well as weight-for-gestational-age *Z* scores on a multicountry scale. The positive exposure–response results for PM_2·5_ and black carbon, in conjunction with measured exposure levels, can be used to inform clean household fuel policy scenarios targeting the reduction of HAP exposures. The results provide further support for continuing efforts to reduce HAP exposure alongside other drivers of low birthweight in LMICs.

Most previous studies that examine the association between HAP exposures and low birthweight have used categorical indicators of exposure based on primary fuel use, with only a handful reporting quantitative exposure–response relationships for PM_2·5_ or smaller^10^, ^11^ or carbon monoxide.^12^, ^13^, ^14^ These exposure–response studies report significant associations between prenatal PM_2·5_ or carbon monoxide exposures and low birthweight, but also report many limitations: small sample sizes, an inability to measure multiple pollutants, and the use of single personal exposure measures during pregnancy and longitudinal kitchen area measurements as proxies of longer-term personal exposure. Randomised control trials of HAP interventions in Nepal,^15^ Nigeria,^12^ and Ghana^16^ have reported null effects from intention-to-treat analyses for effects on birthweight, but exposure–response analyses within these studies have been scarce.^13^ To our knowledge, no studies have examined exposure–response relationships between prenatal black carbon exposures and birthweight.

The multicountry Household Air Pollution Intervention Network (HAPIN) trial was designed to assess health effects after the replacement of biomass cookstoves with liquefied petroleum gas cookstoves in rural Guatemala, India, Peru, and Rwanda, with the goal of reducing HAP in LMICs. The trial was shown to have high fidelity (ie, delivery of the intervention as intended) and adherence to the intervention and also led to a substantial reduction in personal exposure to PM_2·5_ and black carbon exposures during pregnancy.^17^, ^18^ Intention-to-treat analyses on infant birthweight (the first of the primary outcomes reported) were negative, with no significant difference in birthweight between infants born to women who used liquefied petroleum gas cookstoves and those born to women who used biomass cookstoves.^19^ The mean birthweight was 2921 g in the intervention group and 2898 g in the control group, for an adjusted mean difference of 19·5 g (95% CI –10·1 to 49·2). Here we present results from secondary exposure–response analyses for birthweight performed in the HAPIN trial. We hypothesised that high exposure to PM_2·5_, black carbon, and carbon monoxide during pregnancy would result in low birthweight among infants born to women enrolled in the HAPIN trial in each of—and across—the four countries.

## Methods

### Study participants and settings

Participants were pregnant women enrolled in the HAPIN trial, details of which have been published previously^20^, ^21^, ^22^ and are summarised in the trial registration (NCT02944682). The specific study areas in each country (Jalapa Municipality, Guatemala; Villupuram and Nagapatinam districts of Tamil Nadu, India; Department of Puno, Peru; and Eastern Province, Rwanda) were selected based on a high prevalence of cooking with biomass, low background ambient PM_2·5_ concentrations, and acceptable field feasibility as assessed during an 18-month period of planning and formative research.^23^, ^24^ Between March, 2018, and February, 2020, we recruited 3200 (800 per country) non-smoking women who were pregnant, between 18 and 35 years of age, 9 weeks to less than 20 weeks of gestation (determined via ultrasound), and who used biomass as a primary fuel. In accordance with the trial protocol, half of the participants in each country were randomly assigned to an intervention group that received a liquefied petroleum gas stove and a continuous supply of liquefied petroleum gas fuel following enrolment and throughout their pregnancy, whereas the remaining participants acted as controls and continued to rely chiefly on solid biomass for cooking.

The study protocol was reviewed and approved by institutional review boards or ethics committees at: Emory University, Atlanta, GA, USA (00089799), Johns Hopkins University, Baltimore, MD, USA (00007403), Sri Ramachandra Institute of Higher Education and Research, Chennai, India (IEC-N1/16/JUL/54/49), the Indian Council of Medical Research–Health Ministry Screening Committee, New Delhi, India (5/8/4-30/(Env)/Indo-US/2016-NCD-I), Universidad del Valle de Guatemala, Guatemala City, Guatemala (146-08-2016/11-2016), the Guatemalan Ministry of Health National Ethics Committee, Guatemala City, Guatemala (11-2016), A B PRISMA, Lima, Peru, the London School of Hygiene & Tropical Medicine, London, UK (11664-5), the Rwandan National Ethics Committee, Kigali, Rwanda (357/RNEC/2018), and Washington University, St Louis, MO, USA (201611159).

### Personal exposure monitoring during pregnancy

Prenatal personal exposure monitoring protocols and results have been described previously.^22^, ^25^ Briefly, at each study site, women who were pregnant participated in three 24-h personal exposure assessments, once at baseline (<20 weeks of gestation) and twice after randomisation (at 24–28 weeks and 32–36 weeks of gestation). During each session, women wore customised vests or aprons fitted so that the instrumentation was situated close to their breathing zone. PM_2·5_ monitoring was performed using the Enhanced Children's MicroPEM (RTI International, Research Triangle Park, NC, USA), which collects gravimetric samples on pre-weighed 15 mm Teflon filters (Measurement Technologies Laboratories, Minneapolis, MN, USA) using a 2·5 μm impactor at a flow rate of 0·3 L per minute and real-time nephelometric data.^26^ Black carbon was estimated post-sampling on the Enhanced Children's MicroPEM filters using the SootScan Model OT-21 Optical Transmissometer (Magee Scientific, Berkeley, CA, USA). Carbon monoxide monitoring was done using the Lascar EL-CO-USB-300 DataLogger (Lascar Electronics, Erie, PA, USA). Participants were instructed to always wear the vest or apron during the 24-h measurement period, except when sleeping, bathing, or when conducting other activities during which the equipment could not be safely worn. During these times, they were instructed to keep the vest or apron nearby. Additionally, data were collected on sociodemographic and household characteristics and activity patterns that might influence exposure.

Procedures for assuring data quality, weighing filters, and estimating missing gravimetric data based on nephelometry have been described previously.^25^ Briefly, gravimetric data quality assurance involved a combination of threshold values for flow rates, inlet pressure, and sampling duration, as well as visual inspection of damaged filters by the room technicians doing the weighing. In cases for which nephelometric but not gravimetric data were available, PM_2·5_ exposure was estimated based on nephelometric data, using an instrument-specific regression coefficient for the association between nephelometric and gravimetric data for that specific Enhanced Children's MicroPEM instrument as described previously.^25^ Carbon monoxide quality assurance protocols included calibrations with zero air and span gas and a visual inspection system similar to what was applied in the Ghana Randomized Air Pollution and Health Study (GRAPHS)^27^ in Ghana.

For exposure–response analyses, gestational exposures were defined for the intervention group as the average of the pre-intervention and post-intervention exposures, weighted by the amount of gestational time spent in each period. The pre-intervention period exposure was estimated using the baseline measurement, whereas the post-intervention exposure was estimated using one or both personal measurements done after the intervention. This method allowed for exposure changes resulting from the introduction of the intervention to be weighted according to the length of time participants had the intervention during gestation. An unweighted average of the baseline and other available (1–2) gestational period measurements was used for controls, as they continued using biomass as the primary cooking fuel throughout gestation.

### Birthweight outcome measurements

Following a standard protocol, birthweight was measured within 24 h of birth by a trained field worker or nurse using a Seca 334 mobile digital baby scale. Neonates were weighed naked to the nearest 10 g and duplicate measurements were recorded on tablet-based REDCap forms. If the first two measured birthweights differed by more than 10 g, a third measurement was taken. The average of the measurements was used in the data analysis. Neonates were typically assessed at the health facilities where they were born. Each scale was calibrated weekly in the field offices before deployment using standard 5 lb and 10 lb weights; scales not within a 2·5% SD of the standard weight were recalibrated. When we were unable to reach the neonate during the prescribed 24-h window—due mainly to COVID-19 restrictions or critically ill neonates admitted to intensive care units or referral hospitals—we used measurements provided by the facility, if available, but conducted sensitivity analyses to compare results.

As gestational age is a potential mediator in the causal pathway between HAP exposure and birthweight, we did not adjust for it in the exposure–response models; had we done so, its inclusion would not allow estimation of the total effect of exposure.^28^ However, we additionally estimated *Z* scores for weight adjusted by gestational age defined using INTERGROWTH tables as a secondary analysis. These weight-for-gestational-age *Z* scores were derived by subtracting off the standard INTERGROWTH sex-specific weight for a given gestational age and dividing by the INTERGROWTH SD of that weight. Measurements were considered invalid if the gestational age at birth was greater than 300 days or if the birthweight-for-gestational-age *Z* score did not fall between –6 and 5.

### Statistical analysis

The statistical analysis plan was agreed upon in advance and published with the trial registration before unblinding. Analyses were independently replicated by a member of the HAPIN Investigators team (LM). Exposure–response analyses were modelled separately for each pollutant (PM_2·5_, black carbon, and carbon monoxide) and birthweight or birthweight-for-gestational-age *Z* score.

Covariate selection for models was guided by a directed acyclic graph (appendix p 8). A minimal set of potential confounders or strong risk factors (eg, neonate sex) were identified in systematic reviews of birthweight,^3^, ^6^ and from previous studies of HAP and birthweight.^10^, ^11^, ^12^, ^27^, ^29^ We used 5% change-in-estimate methods as outlined by Greenland^30^ to evaluate and determine covariates included in the model. Final models included the following covariates: mother's age (categorical: <20, 20–24, 25–29, or 30–35 years), nulliparity (categorical: yes or no), diet diversity (categorical: low, medium, or high), food insecurity score (categorical: secure, mildly secure [1, 2, or 3], or moderately [4, 5, or 6] or severely [7 or 8] secure), baseline BMI (continuous), mother's education (categorical), neonate sex (categorical), baseline haemoglobin concentration (continuous), and exposure to second-hand smoke (categorical: yes or no). We also included a variable for ten geographical randomisation strata (one in Rwanda, one in Guatemala, two in India, and six in Peru). We created a category of missing for women with missing BMI or haemoglobin data so that they were not excluded from the analysis.

For both birthweight and *Z* scores, we first fitted linear models with different exposure metrics (ie, linear and log linear). We then evaluated non-linear categorical (quartile modes), as well as quadratic, two-piece linear, and restricted cubic spline model with three knots^31^ models, and assessed model fit using Akaike's Information Criterion. The knots for the two-piece spline were chosen based on Akaike's Information Criterion (using quartile cut points initially and then narrowing down), while knots for restricted cubic splines were placed at the 5th, 50th, and 75th percentiles of exposure. We also used thin plate smoothing splines via generalised additive models, with penalisation determined by generalised cross-validation scores, using R package mgcv. We also examined effect modification by country, as well as by neonate sex, via interaction terms between our exposure metrics and these variables.

We did not run multi-pollutant models because the two pollutants that showed some effect on birthweight, black carbon, and PM_2·5_ were highly correlated indicating that the inclusion of both in a model would diminish the effect of each (Spearman coefficient of 0·79). The third pollutant, carbon monoxide, showed no effect on birthweight and its addition to a multi-pollutant model would have had little or no effect.

### Role of the funding source

The funders were not involved in the study design, data collection, data analysis, or interpretation of the data, or the decision to submit the paper for publication.

## Results

3200 women were enrolled in the study, of whom five were determined to be ineligible after randomisation and exited the study. After accounting for miscarriages, stillbirths, and withdrawals, 3195 pregnancies yielded 3060 livebirths (table 1). Of these, 3018 had valid birthweights (others had birthweights measured outside the 24-h window or the study team was unable to obtain any birthweight measurement; appendix p 7). 16 additional births were excluded because of a gestational age of more than 300 days; weight-for-gestational-age *Z* scores are unavailable in the INTERGROWTH database beyond 300 days of gestation. 3002 mother–child pairs were thus eligible for inclusion in exposure–response analyses. These were further restricted by the availability of exposure data for each of the three pollutants of interest. 18 participants were missing BMI data and 26 were missing haemoglobin data but were included in the analyses as part of the category for missing BMI or haemoglobin data.

**Table 1:** Summary of the number of observations used in the exposure–response analysis

	**Number of pregnant women enrolled**	Number with valid birthweights*	**Number with valid PM**_2·5_**exposure measures**	**Number with valid black carbon exposure measures**	**Number with valid carbon monoxide exposure measures**
Guatemala	800	750	703	677	727
India	799	773	710	698	735
Peru	798	730	609	567	600
Rwanda	798	749	695	618	710
Overall	3195	3002 (94%)	2717 (91%)†	2560 (85%)†	2772 (92%)†

Data are n or n (%).

* Women whose neonates had valid birthweights, excluding those whose birth with gestational age was greater than 300 days (*Z* scores unavailable from the INTERGROWTH database).

† Percentage estimates obtained using 3002 as denominator.

The mean age was 25·36 years (SD 4·46; n=3002), with 1151 (38%) of 3002 participants reporting nulliparity (table 2). The average gestational age at recruitment was 15·3 weeks (SD 3·1). 987 (33%) of 3002 women had secondary or higher levels of education. India had the lowest BMI, haemoglobin, and diet diversity scores, whereas Peru had the highest. India had the highest proportion of smokers in the household. Mobile phone ownership was uniformly high across all countries.

**Table 2:** Trial-wide and country-specific maternal characteristics

		**Guatemala (n=750)**	**India (n=773)**	**Peru (n=730)**	**Rwanda (n=749)**	**Overall (n=3002)**
Age, years						
	<20	115 (15%)	122 (16%)	93 (13%)	46 (6%)	376 (13%)
	20–24	303 (40%)	373 (48%)	261 (36%)	187 (25%)	1124 (37%)
	25–29	221 (29%)	223 (29%)	232 (32%)	280 (37%)	956 (32%)
	30–35	111 (15%)	55 (7%)	144 (20%)	236 (32%)	546 (18%)
Gestational age at recruitment, weeks		14·3 (3·0)	16·0 (3·0)	15·7 (3·3)	15·4 (2·8)	15·3 (3·1)
Nulliparous						
	Yes	213 (28%)	442 (57%)	278 (38%)	218 (29%)	1151 (38%)
	No	537 (72%)	337 (44%)	448 (61%)	529 (71%)	1851 (62%)
Highest level of education completed						
	No formal education or some primary school	358 (48%)	275 (36%)	32 (4%)	316 (42%)	981 (33%)
	Primary school or some secondary school	298 (40%)	219 (28%)	224 (31%)	299 (40%)	1040 (35%)
	Secondary, vocational, or some university	100 (13%)	279 (36%)	474 (65%)	134 (18%)	987 (33%)
Height, cm		148 (5·3)	151 (5·6)	152 (4·5)	156 (5·8)	152 (6·2)
BMI, kg/m^2^		23·7 (3·3)	19·7 (3·1)	26·0 (3·5)	23·4 (3·4)	23·2 (4·1)
Haemoglobin, g/dL		12·7 (1·04)	10·3 (1·2)	14·0 (1·2)	12·4 (1·5)	12·4 (1·9)
Minimum dietary diversity score						
	Low (<4)	514 (69%)	600 (78%)	73 (10%)	505 (67%)	1692 (56%)
	Medium (4–5)	206 (27%)	149 (19%)	403 (55%)	208 (28%)	966 (32%)
	High (>5)	30 (4%)	24 (3%)	254 (35%)	35 (5%)	343 (11%)
Household food insecurity score						
	Food secure	415 (55%)	628 (81%)	378 (52%)	276 (37%)	1697 (57%)
	Mild (1, 2, or 3)	238 (32%)	108 (14%)	251 (34%)	212 (28%)	809 (27%)
	Moderate (4, 5, or 6) or severe (7 or 8)	88 (12%)	33 (4%)	91 (12%)	243 (32%)	455 (15%)
Number of people sleeping in the house		5·1 (2·6)	3·7 (1·5)	4·5 (1·7)	3·4 (1·4)	4·3 (2)
Someone in the household smokes						
	Yes	39 (5%)	244 (32%)	7 (1%)	28 (4%)	318 (11%)
	No	711 (95%)	529 (68%)	722 (99%)	719 (96%)	2681 (89%)
Owns household assets						
	Colour television	344 (46%)	577 (75%)	470 (64%)	98 (13%)	1489 (50%)
	Radio	283 (38%)	105 (14%)	540 (74%)	420 (56%)	1348 (45%)
	Mobile phone	687 (92%)	635 (82%)	699 (96%)	594 (79%)	2615 (87%)
	Bicycle	94 (13%)	120 (16%)	278 (38%)	229 (31%)	721 (24%)
	Bank account	186 (25%)	695 (90%)	172 (24%)	221 (30%)	1274 (42%)

Data are n (%) or mean (SD). Descriptive statistics summary based on 3002 pregnant women included in the final analyses, which includes women with livebirths, valid birthweights, and gestational age at birth less than 300 days.

We obtained 2717 valid 24-h prenatal personal PM_2·5_ exposure measurements, 2560 black carbon measurements, and 2772 carbon monoxide measurements (appendix p 4). Mean weighted exposures during pregnancy were 92·2 μg/m^3^ (SD 83·9) for PM_2·5_, 10·0 μg/m^3^ (7·4) for black carbon, and 2·0 ppm (2·9) for carbon monoxide. PM_2·5_ and black carbon exposures were highly correlated (Spearman's p=0·79), but correlations between exposure to PM_2·5_ and carbon monoxide (p=0·34) as well as black carbon and carbon monoxide (p=0·39) were relatively weak. The intervention resulted in reductions in exposure. Post-intervention mean personal PM_2·5_ was 24·0 μg/m^3^ in the intervention group and 70·7 μg/m^3^ in the control group. Similar reductions of exposure were seen for black carbon (2·8 μg/m^3^ *vs* 9·6 μg/m^3^) and carbon monoxide (0·2 ppm *vs* 1·1 ppm).

Details on exposure settings and additional sociodemographic characteristics are reported elsewhere.^25^ Missing exposure data were largely due to equipment failure and were likely to be missing at random.^25^

**Figure 1:**
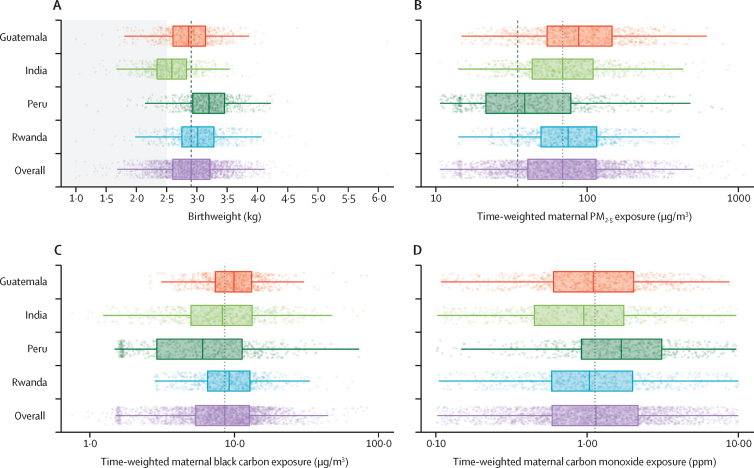
Distribution of (A) birthweight and time-weighted (B) PM₂_·_₅, (C) black carbon, and (D) carbon monoxide The corresponding numeric data are provided in the appendix (p 4). Results are presented separately for each study site and in combination for the entire trial. Dots are individual datapoints. X-axes are log transformed in panels B, C, and D. Thick solid lines inside the boxes are the medians. The lower and upper hinges (ie, the ends of the boxes) correspond to the 25th and 75th percentiles. The whiskers (ie, the lines beyond the boxes) extend from the hinge to 1·5 IQR. The panel-wide dotted vertical lines are study-wide medians. In panel A, the shaded area indicates low birthweight (<2500 g). In panel B, the dashed line is the WHO interim target level one annual guideline value of 35 μg/m^3^.

The mean birthweight of liveborn neonates was 2909 g (SD 471) with mean gestational age at delivery of 39·3 weeks (1·5); 163 (5·3%) of 3002 births were classified as preterm and 531 (17·7%) as low birthweight (figure 1). Mean birthweight was 2921 g (SD 474·3) in the intervention group and 2898 g (467·9) in the control group, a difference of 23 g (95% CI –10·1 to 49·2).

In linear models, an interquartile increase in gestational exposure for PM_2·5_ (74·51 μg/m^3^) was associated with a change in birthweight of –14·8 g (95% CI –28·7 to –0·8) and for black carbon (7·30 μg/m^3^) –21·9 g (–37·7 to –6·1; table 3). For weight-for-gestational-age *Z* scores, the same exposure increases were associated with a decrease of 0·03 (95% CI –0·06 to 0·00) and 0·05 (–0·08 to –0·01) SDs, respectively (table 4). No associations were apparent between carbon monoxide exposures and birthweight in the linear models or between any of the measured pollutants and low birthweight prevalence. Quartile analyses (appendix p 2) showed that the decrease in birthweight and *Z* scores were not monotonic for PM_2·5_, whereas decreases were monotonic for *Z* scores but not birthweight for black carbon.

**Table 3:** Change in birthweight for an IQR increase in PM_2·5_, black carbon, and carbon monoxide

	**Model type**	**Estimate (95% CI)**	**p value**	**Akaike's Information Criterion**
**Adjusted associations**				
PM_2·5_	Linear	−14·8 (−28·7 to −0·8)	0·038	40 211
PM_2·5_	Log linear	−11·2 (−33·6 to 11·2)	0·327	40 215
Black carbon	Linear	−21·9 (−37·7 to −6·1)	0·007	37 876
Black carbon	Log linear	−19·2 (−40·1 to 1·7)	0·071	37 880
Carbon monoxide	Linear	−3·1 (−12·1 to 5·8)	0·499	41 017
Carbon monoxide	Log linear	10·6 (−7·2 to 28·4)	0·245	41 017
Crude associations*				
PM_2·5_	Linear	−19·5 (−33·8 to −5·2)	0·008	40 458
PM_2·5_	Log linear	−21·6 (−44·4 to 1·3)	0·064	40 462
Black carbon	Linear	−25·5 (−41·7 to −9·4)	0·002	38 070
Black carbon	Log linear	−25·2 (−46·4 to −4·0)	0·019	38 074
Carbon monoxide	Linear	−3·5 (−12·7 to 5·7)	0·451	41 268
Carbon monoxide	Log linear	8·2 (−10·2 to 26·6)	0·384	41 268

Both models were adjusted for mother's education, baseline BMI, nulliparity, diet diversity, food insecurity score, second-hand smoke, baseline haemoglobin, age, neonate sex, and ten randomisation strata. For the linear model, the IQR for PM_2·5_ was 74·51, for black carbon was 7·30, and for carbon monoxide was 1·68. On the log scale, IQRs for were 1·04 for PM_2.5_, 0·85 for black carbon, and 1·40 for carbon monoxide.

* Estimates for change per IQR for crude models were adjusted only for randomisation strata.

**Table 4:** Change in weight-for-gestational age *Z* scores with an IQR increase in PM_2·5_, black carbon, and carbon monoxide

	**Model**	**Estimate (95% CI)**	**p value**	**Akaike's Information Criterion**
**Adjusted associations**				
PM_2·5_	Linear	−0·03 (−0·06 to 0·00)	0·380	7021
PM_2·5_	Log linear	−0·04 (0·09 to 0·01)	0·100	7023
Black carbon	Linear	−0·05 (−0·08 to −0·01)	0·006	6591
Black carbon	Log linear	−0·06 (−0·10 to −0·01)	0·019	6593
Carbon monoxide	Linear	−0·003 (−0·023 to 0·017)	0·780	7215
Carbon monoxide	Log linear	0·02 (−0·02 to 0·06)	0·239	7214
Crude associations*				
PM_2·5_	Linear	−0·04 (−0·07 to −0·01)	0·013	7161
PM_2·5_	Log linear	−0·06 (−0·11 to −0·01)	0·020	7162
Black carbon	Linear	−0·05 (−0·09 to −0·02)	0·003	6708
Black carbon	Log linear	−0·06 (−0·11 to −0·02)	0·007	6710
Carbon monoxide	Linear	−0·003 (−0·024 to 0·017)	0·739	7342
Carbon monoxide	Log linear	0·02 (−0·02 to 0·06)	0·323	7341

All models adjusted for mother's education, baseline BMI, nulliparity, diet diversity, food insecurity score, second-hand smoke, baseline haemoglobin, age, neonate sex, and ten randomisation strata. For the linear model, the IQR for PM_2·5_ was 74·51, for black carbon was 7·30, and for carbon monoxide was 1·68. On the log scale, IQRs were 1·04 for PM_2.5_, 0·85 for black carbon, and 1·40 for carbon monoxide.

* Estimates for change in IQR from a crude model adjusting only for randomisation strata.

**Figure 2:**
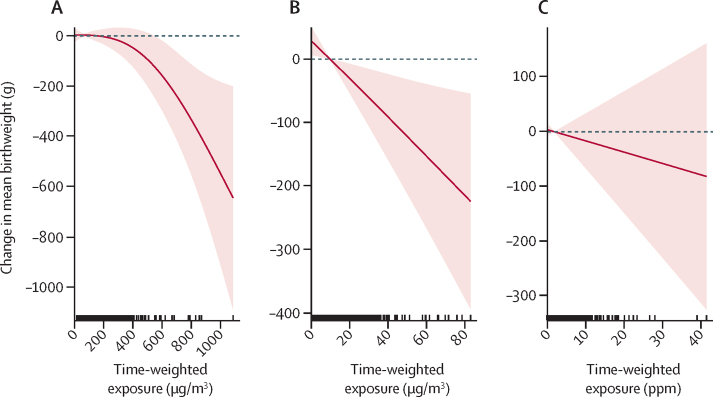
Exposure–response relationships between birthweight and prenatal (A) PM_2·5_, (B) black carbon, and (C) carbon monoxide personal exposures Dashed lines correspond to the 95% CI and vertical dashes along the x-axis are observed measurements.

**Figure 3:**
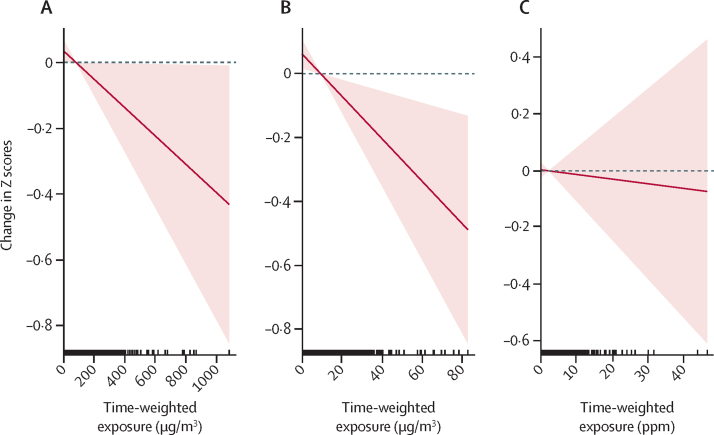
Exposure–response relationships between weight-for-gestational age *Z* scores and prenatal (A) PM_2·5_, (B) black carbon, and (C) carbon monoxide personal exposures Dashed lines correspond to the 95% CI and vertical dashes along the x-axis are observed measurements.

Evaluation of different models indicated that the linear was appropriate to model the relationships between the birthweight outcomes and black carbon. For PM_2·5_, however, a quadratic (non-linear) fit was better suited to the birthweight outcome (appendix pp 3, 8), with a positive linear coefficient (0·2325) and a negative quadratic coefficient (–0·009), indicating an initial increase in birthweight with higher PM₂_·_₅ followed by a subsequent decrease at the higher exposures. Both categorical and cubic spline models supported this relationship (appendix p 8). Linear models fit best for black carbon for both birthweight and *Z* scores, as well as PM_2·5_ and *Z* scores. Smoothed exposure–response curves for PM_2·5_ and black carbon and birthweight and weight-for-gestational-age *Z* scores can be seen in figure 2 and figure 3.

Trends for full-term births (2839 of 3002, 94% of births) were similar to trends for all births (appendix p 3). No statistically significant interactions were observed with neonate sex, but female births showed a larger effect than male births for birthweight, and for *Z* scores (appendix p 3). Trends were reasonably consistent across countries for the association between PM_2·5_ and black carbon with both birthweight and *Z* scores (appendix p 5). We also ran separate models for our three exposure measurements during gestation—ie, for baseline, midpoint, and end of gestation measurements (these corresponding roughly to early second trimester, end of second trimester, and end of third trimester). These models, for both birthweight and *Z* score, showed no pattern whereby early or later exposures had stronger effects on the outcome (appendix p 6). Indeed all time-specific exposure–response coefficients were weaker than those coefficients using average exposure. This outcome might occur because single measurements involve more measurement error than average exposure across gestation, biasing results to the null.

## Discussion

The findings of this study suggest that reducing prenatal HAP exposure could yield modest potential benefits for birthweight that are not consistent across all pollutants. To our knowledge, ours is the first study reporting on exposure–response relationships between gestational black carbon exposures from HAP and birthweight. Notably, a 7·3 μg/m^3^ reduction in prenatal black carbon exposure was associated with an increase in birthweight of about 22 g, which could have positive implications for populations with a high prevalence of low birthweight. The results from the recently reported intention-to-treat analyses in the HAPIN trial^19^ were negative but showed a 20 g higher birthweight for the intervention group than the control group (albeit not statistically significant). The secondary exposure–response analysis from the trial reported here is consistent with these results, showing a decrease in birthweight for those with higher exposure.

Only three previous studies have published quantitative exposure–response results for birth outcomes in relation to HAP exposure, focusing on PM_2·5_ or carbon monoxide. In a cohort of 239 pregnant women in Tanzania, there was a negative association between carbon monoxide exposure and newborn birthweight, but results were not statistically significant.^32^ The Tanzania study also reported a 150 g (95% CI –300 to 0) reduction in birthweight per 23·0 μg/m^3^ increase in PM_2·5_. The second study, among 1285 women in the Tamil Nadu region of India, reported a 4 g (95% CI 1·08–6·76) decrease in birthweight and a 2% increase in the prevalence of low birthweight (0·05–4·1) for each 10 μg/m^3^ increase in kitchen area PM_2·5_ measured during pregnancy.^10^ The third study, conducted as part of the GRAPHS trial in Ghana,^13^ observed the effects of carbon monoxide on birthweight, birth length, and gestational age that were modified by placental malarial status. Among infants from pregnancies without evidence of placental malaria, each 1 ppm increase in carbon monoxide was associated with reduced birthweight (−53·4 g, 95% CI −84·8 to −21·9), birth length (−0·3 cm, −0·6 to −0·1), gestational age (−1·0 days, −1·8 to −0·2), and weight-for-gestational-age *Z* score (−0·08 SD, −0·16 to −0·01). These associations were not observed in pregnancies with evidence of placental malaria. PM_2·5_ measurements were, however, scarce in the GRAPHS trial and no association between PM_2·5_ exposure and birthweight was observed.

The negative associations between PM_2·5_ exposures and birthweight in our study are consistent with previous studies, but at the lower end of reported estimates. In contrast, the lack of an association between prenatal carbon monoxide exposure and birthweight was unexpected. However, this finding is not entirely surprising as the correlations between PM_2·5_ and carbon monoxide have not been uniform across HAP settings. A systematic review examining this relationship^33^ found inconsistent correlation with slightly stronger correlation among exclusive biomass users relative to mixed fuel users (R^2^=0·29 *vs* 0·18). The relatively modest correlations between either PM_2·5_ or black carbon and carbon monoxide observed in our study could be attributable to our study conditions of exclusive biomass and liquefied petroleum gas use.

Although the kind of effect on birthweight seen by decreasing pollutant exposure (eg, an increase of 20 g with a reduction of pollutant equal to the IQR) might not be clinically significant for an individual, at a population level shifting the distribution of birthweight by a small amount can have important benefits for population health.^34^ We estimate that a shift from average HAP levels in LMICs to the WHO interim target level one of 35 μg/m^3^ of PM_2·5_ could decrease infant mortality by about 170 000 deaths per year (appendix p 9).

Trials of cookstove interventions to improve birth outcomes have had mixed outcomes: an improved biomass cookstove in a cohort of 174 infants in Guatemala was associated with 89 g (95% CI –27 to 204) higher birthweight in adjusted analysis,^14^ and a clean-burning ethanol stove intervention in Nigeria was associated with 128 g (20 to 236) higher birthweight among 258 infants in adjusted analysis.^12^ Meanwhile, neither an improved biomass nor a liquefied petroleum gas stove improved birth outcomes in two linked trials covering almost 3000 individuals in southern Nepal.^15^ These trials have not reported quantitative exposure–response relations. In the GRAPHS trial,^13^ although there was a significant exposure–response relationship between carbon monoxide exposures and birthweight, neither prenatally introduced liquefied petroleum gas nor improved biomass cookstoves improved birthweight. The investigators in all previous trials hypothesised that these findings are perhaps due to lower-than-expected exposure reductions in the intervention groups.

The HAP exposure levels associated with biomass use (such as at baseline and in the control group) in our study are at the lower end of what has been reported in previous trials, with the possible exception of the GRAPHS trial. On the basis of pilot phase exposure reductions^23^, ^24^ and estimated supra-linear exposure–response relationships for HAP and birthweight,^35^ we hypothesised that the levels observed during pilot work implied that exposure reductions would occur on the steep part of the response curve for birthweight. Given the relative paucity of studies on quantitative exposure–response analyses for HAP based on personal exposures, the shape of the exposure–response curve could possibly be different than what was previously estimated. Our study contributes important information regarding this relationship based on high-quality personal HAP exposure and birthweight measurements from four diverse settings that can inform future development of pooled exposure–response coefficients spanning the range of experienced HAP exposures and could inform future exposure–response curves that integrate across air pollution sources.

Following our original statistical analysis plan for the exposure–response analyses, we did not adjust p values for multiple comparisons. We note, however, that if we had used a Benjamini-Hochberg false discovery rate adjustment for six comparisons (eg, two outcomes and three pollutants), we would have used a p value of 0·03 instead of 0·05 as a significance level, and the inverse associations between black carbon and both birthweight and *Z* scores would have remained significant.^36^

There were several limitations to our study. We note that other unmeasured factors including placental malaria, water and sanitation, and nutritional deficiencies could have outweighed the effects of HAP on birthweight outcomes. We measured personal exposure only three times during pregnancy. Although this number is more than most studies, such measurements undoubtebly involve some error. However, as this error is most likely to be non-differential (ie, not differing by birthweight or *Z* score), it is likely to have biased our exposure–response findings to the null.

In this study population drawn from diverse sociodemographic settings across four countries, exposure to HAP—particularly to black carbon and to a lesser extent to PM_2·5_—during pregnancy was associated with reduced birthweight and weight-for-gestational-age *Z* scores. To our knowledge, ours is the first study reporting on exposure–response relationships between gestational black carbon exposures from HAP and birthweight. The association, although modest, provides strong support for continuing efforts to address HAP exposures alongside other drivers of impaired fetal growth in LMICs.

## Data sharing

De-identified individual participant data, as well as the study protocol, statistical analysis plan, informed consent form, and analytics code will be available to anyone who wishes to access the data for any purpose, from 6 months post publication, via DataVerse.

For more on **INTERGROWTH** see https://intergrowth21.tghn.org

For more on **DataVerse** see https://dataverse.unc.edu/dataverse/Emory

## Declaration of interests

We declare no competing interests.
