# Supplementary material for: Exposure–response relationships for personal exposure to fine particulate matter (PM2·5), carbon monoxide, and black carbon and birthweight: an observational analysis of the multicountry Household Air Pollution Intervention Network (HAPIN) trial
Source: Lancet Planet Health. 2023 May 8;7(5):e387–96. doi: 10.1016/S2542-5196(23)00052-9 (PMC10186177; doi:10.1016/S2542-5196(23)00052-9)
Supplement: Supplementary appendix [file mmc1.pdf]

THE LANCET
Planetary Health

Supplementary appendix

This appendix formed part of the original submission and has been peer reviewed. We post it as supplied by the authors.

Supplement to: Balakrishnan K, Steenland K, Clasen T, et al. Exposure–response relationships for personal exposure to fine particulate matter ($PM_{2·5}$), carbon monoxide, and black carbon and birthweight: an observational analysis of the multicountry Household Air Pollution Intervention Network (HAPIN) trial. *Lancet Planet Health* 2023; **7:** e387–96.

Supplementary Material

Table S1: Change in birthweight for by quartiles of exposure	2
Table S2: Change in z-scores by quartiles of exposure	2
Table S3. AICs for different models for birthweight and z-score, in relation to $PM_{2 \cdot 5}$ and BC	3
Table S4. Change in outcome by increase in pollutant IQR for full term births	3
Table S5. Change in outcome by increase in pollutant IQR by infant sex	3
Table S6: Birthweights and pollutant exposures by country and overall	4
Table S7. Linear models for birthweight and three pollutants in each country	5
Table S8. Linear models for weight-for gestational age z-scores and three pollutants in each country	5
Table S9. Exposure-response coefficient using exposure as measured in early, middle, and late gestation	6
Figure S1. CONSORT diagram	7
Figure S2. Graphs of linear, quadratic, 2-piece linear and cubic spline models for $PM_{2 \cdot 5}$ and birthweight	8
Figure S3. Directed acyclic graph (DAG) to guide the selection of confounders for the association between household air pollution exposure and birthweight.	8
Appendix. Potential impact of decrease in HAP on increased birthweight and infant mortality	9

Table S1: Change in birthweight for by quartiles of exposure*

Pollutant	Model	Estimate (95% CI)	p-value	AIC
$PM_{2·5}$	Quartile 1	*reference*		40218
	Quartile 2	-24·9 (-68·9, 19·2)	0·27	
	Quartile 3	-5·2 (-50·1, 39·7)	0·82	
	Quartile 4**	-24·5 (-70·4, 21·4)	0·30	
BC	Quartile 1	*reference*		37885
	Quartile 2	-29·9 (-75·3, 15·5)	0·20	
	Quartile 3	-18·3 (-64·9, 28·3)	0·44	
	Quartile 4**	-39·2 (-86·1, 7·7)	0·10	
CO	Quartile 1	*reference*		
	Quartile 2	44·0 (1·7, 86·2)	0·04	
	Quartile 3	47·4 (4·9, 89·9)	0·03	
	Quartile 4**	16·1 (-27·3, 59·5)	0·47	41015

*All models adjusted for mother's education, baseline BMI, nulliparity, diet diversity, food insecurity score, second-hand smoke, baseline hemoglobin, age, as well as infant sex, and 10 randomization strata.
**Cutpoints for quartiles for $PM_{2·5}$: 40·8, 69·5, and 115·3cutpoints for quartiles for BC: 5·4, 8·6, and 12·7, cutpoints for quartiles for CO: 0·55, 1·13, and 2·23.

Table S2: Change in z-scores by quartiles of exposure*

Pollutant	Model	Estimate (95% CI)	p-value	AIC
$PM_{2·5}$	Quartile 1	*reference*		7024
	Quartile 2	-0·094 (-0·191, 0·002)	0·06	
	Quartile 3	-0·045 (-0·144, 0·054)	0·37	
	Quartile 4**	-0·102 (-0·202, 0·001)	0·05	
BC	Quartile 1	*reference*		6595
	Quartile 2	-0·063 (-0·163, 0·037)	0·21	
	Quartile 3	-0·076 (-0·179, 0·023)	0·14	
	Quartile 4**	-0·137 (-0·240, -0·034)	0·01	
CO	Quartile 1	*reference*		7212
	Quartile 2	0·013 (0·038, 0·225)	0·01	
	Quartile 3	0·097 (0·003, 0·192)	0·04	
	Quartile 4**	0·060 (-0·037, 0·156)	0·23	

*All models adjusted for mother's education, baseline BMI, nulliparity, diet diversity, food insecurity score, second-hand smoke, baseline hemoglobin, age, infant sex, and 10 randomization strata.
**Cutpoints for quartiles for PM2·5: 40·8, 69·5, and 115·3cutpoints for quartiles for BC: 5·4, 8·6, and 12·7, cutpoints for quartiles for CO: 0·55, 1·13, and 2·23.

Table S3. AICs for different models for birthweight and z-score, in relation to $PM_{2·5}$ and BC

Outcome	Exposure	Model	AIC
birthweight	$PM_{2·5}$	linear	40211·7
		Log linear	40215·0
		quadratic	40206·2
		2 piece linear	40207·4
		Cubic spline	40212·4
birthweight	BC	*linear*	37876·6
		Log linear	37880·8
		quadratic	37878·2
		2 piece linear	37877·5
		Cubic spline	37878·2
z-score	$PM_{2·5}$	*linear*	7021·6
		Log linear	7023·2
		quadratic	7023·5
		2 piece linear	7022·9
		Cubic spline	7023·6
z-score	BC	*linear*	6590·7
		Log linear	6592·8
		quadratic	6592·5
		2 piece linear	6592·3
		Cubic spline	6592·5

Table S4. Change in outcome by increase in pollutant IQR for full term births

Outcome	Pollutant and model	Change in outcome (g)	LCL change	UCL change
birthweight	$PM_{2·5}$ linear	-13·315	-27·017	0·395
	BC linear	-19·701	-34·757	-4·645
	CO linear	-3·183	-11·570	5·204
z-score	$PM_{2·5}$ linear	-0·045	-0·075	-0·015
	BC linear	-0·054	-0·089	-0·018
	CO linear	-0·006	-0·025	0·014

Table S5. Change in outcome by increase in pollutant IQR by infant sex

Outcome	Pollutant and model	Change in outcome (g)	LCL change	UCL change
birthweight (male)	$PM_{2·5}$ linear	-2·776	-23·415	17·863
	BC linear	-7·871	-31·385	15·643
	CO linear	-6·144	-19·729	7·441
birthweight (female)	$PM_{2·5}$ linear	-26·674	-46·009	-7·339
	BC linear	-34·745	-56·388	-13·103
	CO linear	-0·081	-12·063	11·901
z-score (male)	$PM_{2·5}$ linear	-0·032	-0·076	0·012
	BC linear	-0·040	-0·090	0·010
	CO linear	-0·010	-0·039	0·020
z-score (female)	$PM_{2·5}$ linear	-0·031	-0·075	0·012
	BC linear	-0·059	-0·108	-0·010
	CO linear	0·002	-0·026	0·029

Table S6: Birthweights and pollutant exposures by country and overall

	Birthweight (grams)			$PM_{2\cdot5}$ (µg/m^3)				BC (µg/m^3)				CO (ppm)			
	**Mean**	**SD**	**N**	**Mean**	**SD**	**Median**	**N**	**Mean**	**SD**	**Median**	**N**	**Mean**	**SD**	**Median**	**N**
India	2591	394	773	94·29	90·90	69·32	710	10·44	8·49	8·24	698	1·49	2·21	0·87	735
Rwanda	3022	439	749	93·50	72·45	75·42	695	10·42	6·38	9·19	618	1·94	2·66	1·04	710
Guatemala	2861	428	750	112·88	83·89	88·62	703	10·90	6·40	9·94	677	1·56	1·64	1·08	727
Peru	3180	410	730	64·37	79·85	38·99	609	7·96	7·71	5·75	567	3·09	4·41	1·76	600
Study-wide	2909	471	2560	92·19	83·85	69·45	2717	10·01	7·39	8·57	2560	1·97	2·90	1·13	2772

Table S7. Linear models for birthweight and three pollutants in each country

Pollutant and model	Country	Exposure-response coefficient	p-value
$PM_{2\cdot5}$ linear model	Guatemala	-0·11	0·55
	India	-0·09	0·57
	Peru	-0·16	0·44
	Rwanda	-0·56	0·01
	Combined	-0·20	0·04
BC linear model	Guatemala	-4·5	0·07
	India	-2·6	0·13
	Peru	-0·9	0·69
	Rwanda	-3·5	0·22
	Combined	-3·0	0·01
CO linear model	Guatemala	-10·6	0·26
	India	3·1	0·61
	Peru	-5·6	0·13
	Rwanda	10·9	0·24
	Combined	-1·8	0·49

Table S8. Linear models for weight-for-gestational age z-scores and three pollutants in each country

Pollutant and model	Country	Exposure-response coefficient	p-value
$PM_{2\cdot5}$ linear model	Guatemala	-0·0005	0·29
	India	-0·0004	0·25
	Peru	-0·0002	0·61
	Rwanda	-0·0006	0·23
	Combined	-0·0004	0·04
BC linear model	Guatemala	-0·125	0·03
	India	-0·006	0·09
	Peru	-0·001	0·76
	Rwanda	-0·005	0·37
	Combined	-0·007	0·01
CO linear model	Guatemala	-0·006	0·79
	India	0·011	0·40
	Peru	-0·010	0·23
	Rwanda	0·009	0·49
	Combined	-0·002	0·78

Table S9. Exposure-response coefficient using exposure as measured in early, middle, and late gestation

	Coefficient	t-statistic	p-value
**Birthweight**			
BC overall avg	-2·9996	7·42	0·0065
BC BL*	-0·856	1·06	0·3044
BC P1*	-1·8725	3·38	0·0661
BC P2*	-1·8086	3·62	0·0571
$PM_{2·5}$ overall avg	-0·1983	4·3	0·0381
$PM_{2·5}$ BL	-0·07	1·15	0·2843
$PM_{2·5}$ P1	-0·0033	0	0·9728
$PM_{2·5}$ P2	-0·0645	0·51	0·4761
CO overall avg	-1·8209	0·46	0·4999
CO BL	2·3535	1·59	0·2076
CO P1	2·1364	0·61	0·4357
CO P2	-3·7919	2·13	0·1448
**Weight-for-gestational age z-scores**			
BC overall avg	-0·0067	7·62	0·0058
BC BL*	-0·0028	2·41	0·1202
BC P1*	-0·004	3·09	0·0786
BC P2*	-0·0043	4·17	0·0411
$PM_{2·5}$ overall avg	-0·0004	4·32	0·0377
$PM_{2·5}$ BL	-0·0002	2·61	0·1063
$PM_{2·5}$ P1	-0·0001	0·19	0·6659
$PM_{2·5}$ P2	-0·0002	0·88	0·3475
CO overall avg	-0·0017	0·08	0·7787
CO BL	0·0064	2·38	0·1226
CO P1	0·0043	0·5	0·4794
CO P2	-0·0073	1·58	0·2089

*BC = black carbon, CO = carbon monoxide

**BL = baseline measurement, P1 and P2 are 2nd and 3rd measurements

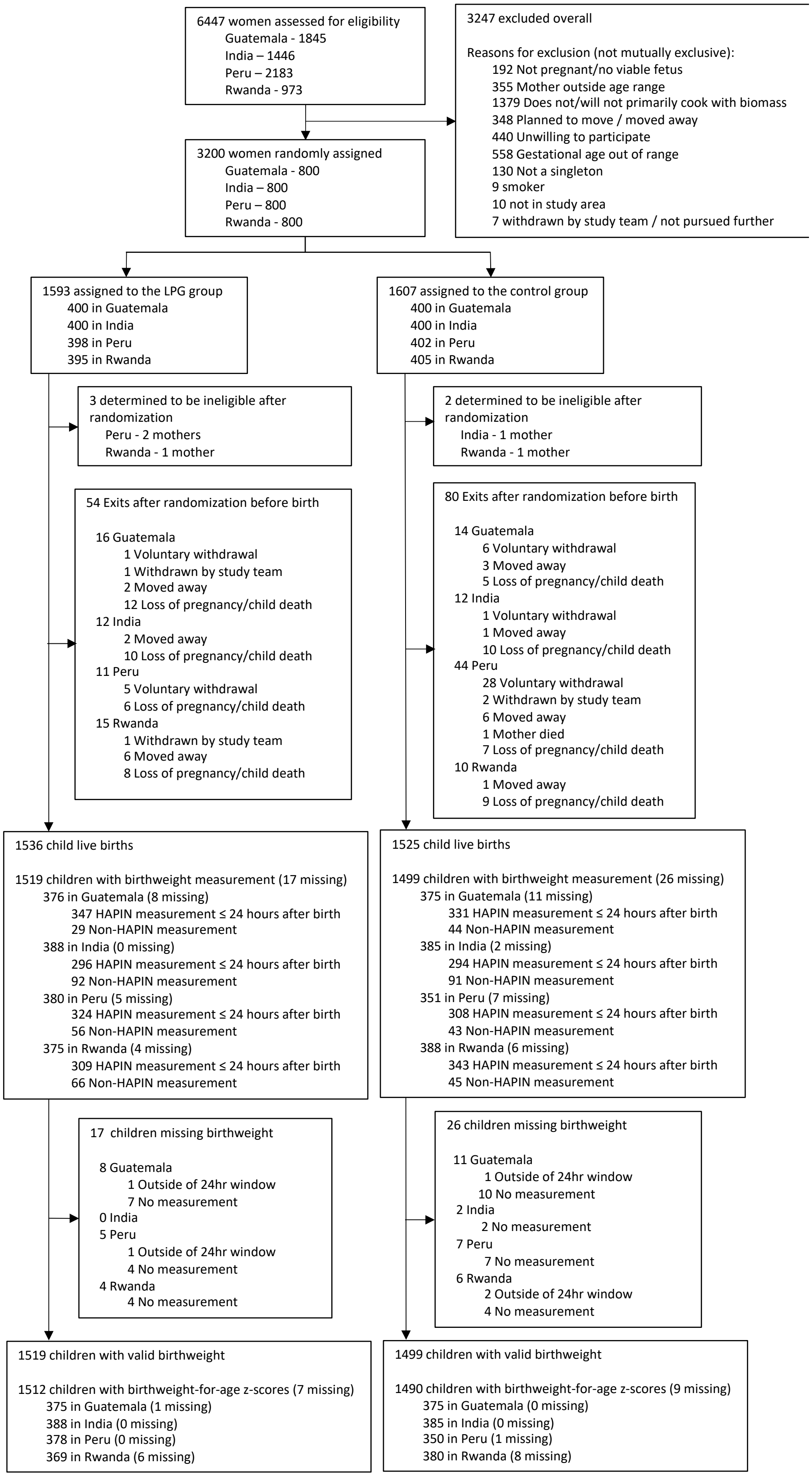

Figure S1. CONSORT diagram

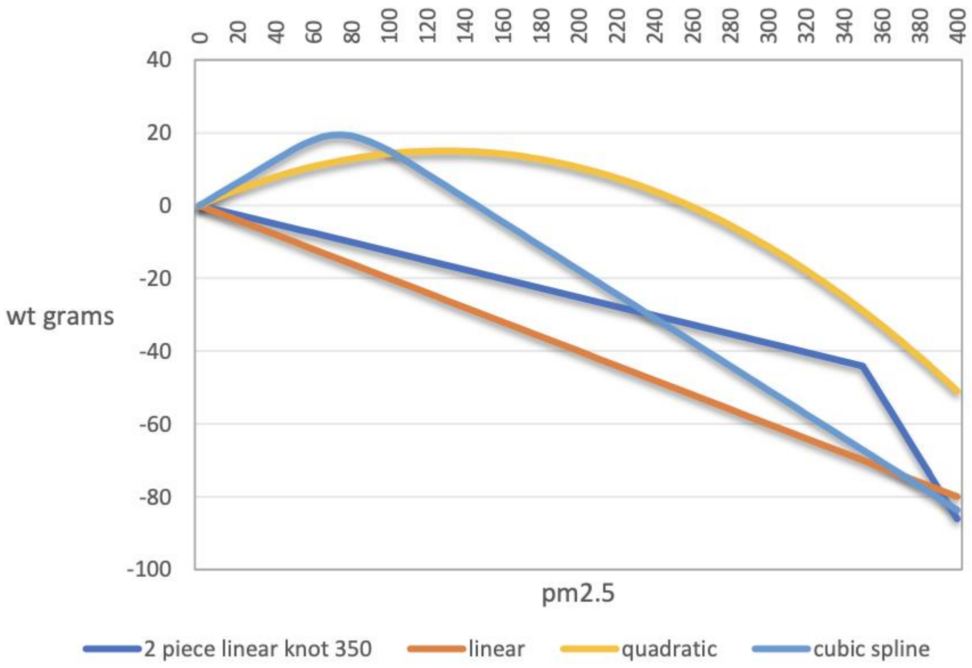

Figure S2. Linear, quadratic, 2-piece linear and cubic spline models for $PM_{2.5}$ and birthweight Linear model AIC 40212; 2-piece spline model knot 350, AIC 40207; Quadratic AIC 40206. Cubic spline model AIC 40212.

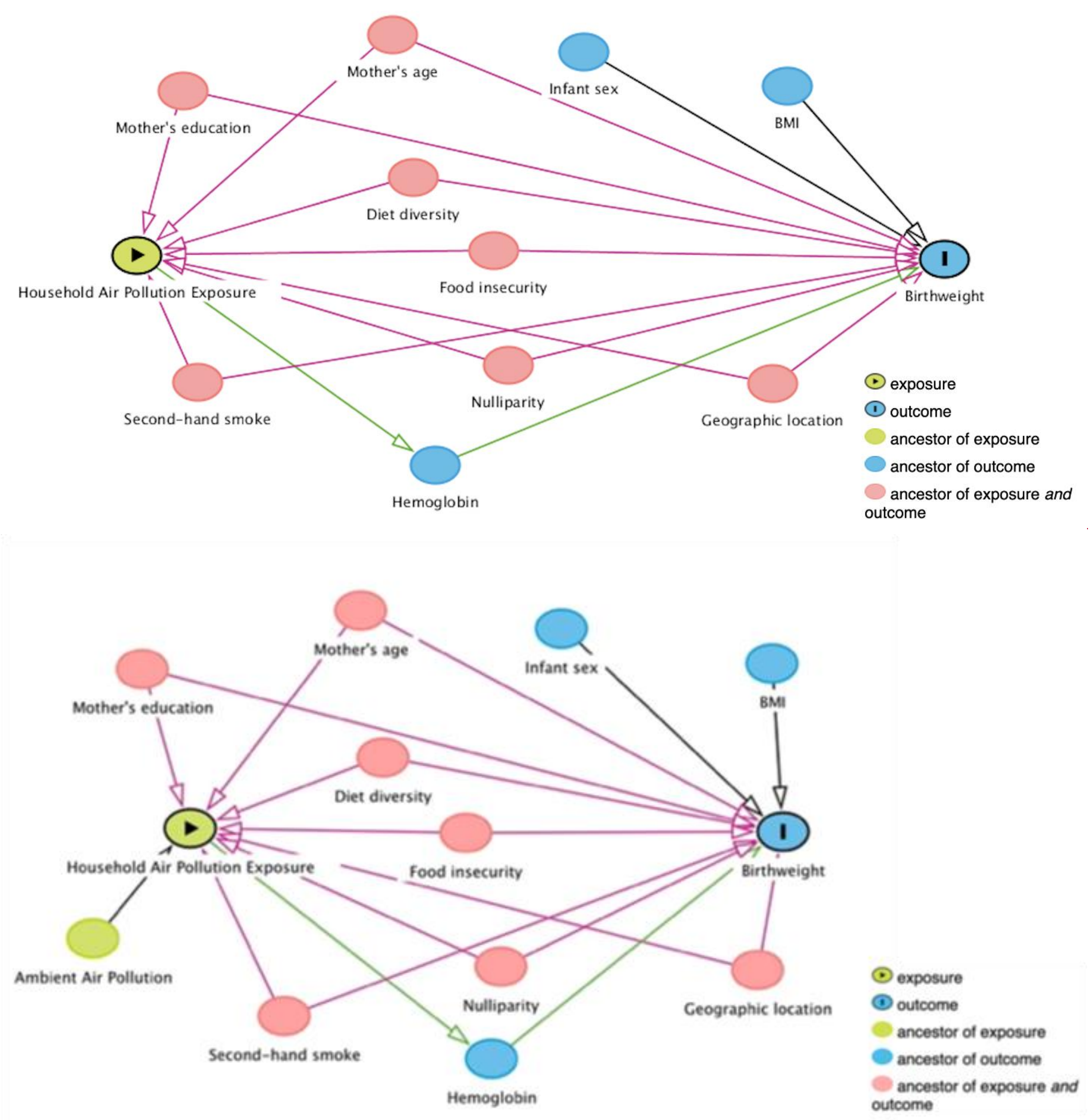

Figure S3. Directed acyclic graph (DAG) of the a priori covariates adjusted in the exposure-response models for the associations between household air pollution exposure and birthweight. Causal path (green line); Biasing path (red line).

Appendix. Potential impact of decrease in HAP on increased birthweight and infant mortality

We found a coefficient of -0.15 birthweight change per unit of $PM_{2.5}$, or 1.5 gm decrease per 10 μg/m^3 increase in $PM_{2.5}$. This translates to about a 20 gm increased in birthweight for a lowering of 1 IQR of $PM_{2.5}$ in our study. Another recent paper (Balakrishnan et al. 2018) of HAP and birthweight found a decrease of 4 gm per 10 μg/m^3

increase in $PM_{2.5}$. Let us then assume that the true effect is about a 2.7 gm decrease in birthweight per 10 µg/m^3, an average of our study and Balakrishnan et al's. 2018 finding. Let us further assume that the average personal exposure to $PM_{2.5}$ among biomass users worldwide is about 270 µg/m^3 (based on reviews by Rehfuess et al. 2014, and Balakrishnan et al. 2014), higher than our study which had a mean of about 150 ug/m3 (Johnson et al 2022). Let us further assume a gas intervention would drop this to 35 µg/m^3, the WHO interim target 1 goal (WHO 2021) and about what we found in our intervention group (Johnson et al 2022). That would mean that the birthweight among biomass users should increase by about 66gms (=24*2.75), with a decrease of $PM_{2.5}$ from 270 µg/m^3 to 35 µg/m^3. While such an increase may not be of clinical significance for individuals, shifting birthweight this much would be expected to decrease infant mortality on a population basis. If we assume that there is a drop of one unit (per 1000) in infant mortality for each 30 gm of birthweight (Wilcox, 2001), and an infant mortality rate of 30/1000 in LMICs (World Bank 2017). an improvement of 66 gm would translate to an approximate drop from 30 to 28/1000 in infant mortality. WHO estimates that there are 2.4 million infant deaths per year (in the first month of life, when most infant deaths occur), the great majority in LMICs (WHO 2020), a 7% decrease translates then, to a decrease of about 168,000 infant deaths per year.

References

Wilcox AJ. On the importance--and the unimportance--of birthweight. Int J Epidemiol. 2001 Dec;30(6):1233-41.

World Bank 2017, http://data.worldbank.org/indicator/SP.DYN.IMRT.IN, last accessed Nov 16, 2022

WHO Global Air Quality Guidelines, 2021, https://apps.who.int/iris/bitstream/handle/10665/345329/9789240034228-eng.pdf, last accesed Nov 16, 2022

Rehfuess E, Pope D, Bruce N, Dherani M, Jagoe K, Naeher L, and Noonan C, Review 6: Impacts of interventions on household air pollution concentrations and personal exposure, WHO Indoor Air Quality Guidelines: Household Fuel Combustion, WHO, Geneva, 2014.

Balakrishnan K, Mehta S, Ghosh S, Johnson M, Brauer B, Zhang J, Naeher L, Smith K, Review 5: Population levels of household air pollution and exposures, WHO Indoor Air Quality Guidelines: Household Fuel Combustion, WHO, Geneva, 2014.

WHO 2020, https://www.who.int/news-room/fact-sheets/detail/levels-and-trends-in-child-mortality-report-2021.
